# Chylous Ascites in a Newborn with Gastroschisis. Case Report

**DOI:** 10.21699/jns.v6i1.428

**Published:** 2017-01-01

**Authors:** Cristian Ruben Zalles-Vidal, Alejandro Peñarrieta-Daher, Daniel Ibarra-Rios, Emilio Fernandez-Portilla, Eduardo Bracho-Blachet

**Affiliations:** 1 Department of Pediatric Surgery (Neonatal Surgery Service), Hospital Infantil de México Federico Gomez, México City.; 2 Department of Neonatology, Hospital Infantil de México Federico Gomez, México City.

**Keywords:** Chylous ascites, Gastroschisis, Treatment, Octreotide

## Abstract

Chylous ascites is a rare disease, only two cases associated with gastroschisis have been published. We report a case treated conservatively with total parenteral nutrition (TPN) and octreotide. We reviewed the literature about management options for the chylous ascites.

## CASE REPORT

A female newborn of an 18-year-old mother with prenatal diagnosis of gastroschisis born with birth weight was 2310 g at 37 weeks of gestation. At first evaluation by the pediatric surgery team, a Gastroschisis Prognosis Score of 1 was given. A ward reduction and suture-less closure was done in the neonatal intensive care unit during the first hours of life without the need of mechanical ventilation. Total parenteral nutrition was started through a PICC line the next day. Enteral feed was started at the 7th day of life, achieving total enteral feeding on the 13th day of life. She presented cholestasis [direct bilirubin (DB) 4 mg/dl] with acholic stools at the second week of life. Hepatobiliary scintigraphy demonstrated an intrahepatic cholestasis pattern with bile passing into the bowel. At 1 month of age abdominal distention with ascites appeared, a paracentesis obtained a milky yellow fluid. On peritoneal fluid analysis, triglycerides were 434mg/dL, proteins 3934mg/dL and high leukocytes count 1870mm3 with 97% monocytes, confirming the diagnosis of chylous ascites. Initial treatment was begun with enteral formula low in triglycerides (Deilem®) and octreotide (2μg/kg/h) without response to treatment. A new paracentesis was required because of abdominal distension with respiratory distress, fluid like the previously was obtained. After the second paracentesis, feces started to have green color and the DB normalized. Octreotide was increased daily by 1μg/kg/hour until 10μg/kg/hour. At the third week of treatment, octreotide was decreased progressively, and enteral feeding was started at 24ml/kg/day with Deilem® supplemented with medium-chain triglycerides (MCT). The patient received this formula for 2 months as an outpatient, afterwards it was then changed to normal formula without any relapse of the chylous ascites during an 8-month follow-up. Growth and development are normal.

## DISCUSSION

This is the third case report of CA associated with gastroschisis. CA is an uncommon disease with high morbidity and mortality in patients who are not treated appropriately and aggressively. Etiology is varied and different per age groups. In the newborn, it is common to find lymphatic malformations or diseases that causes obstruction of bowel lymphatic flow such as intestinal malrotation [1], venous thrombosis or gastroschisis [2,3]. In older children, one can see lymphatic lesions secondary to abdominal surgery and infections such as tuberculosis or parasitosis. In adults, the main causes are neoplasms and trauma (due to hyperextension or flexion of the lymph vessels) [4].

Diagnosis is based on clinical findings. A painless abdominal distention is common because of ascites. Tenderness should guide towards a peritoneal infection. Abdominal distention becomes apparent or increases when the patient start feedings, as seen in our case. When a paracentesis is done the appearance of the peritoneal fluid may vary: it is usually described as a milky fluid with a triglyceride level >100 mg/dL, high leukocytes (sometimes higher than in serum) with predominance of lymphocytes and protein level is increased. These findings could change if the patient is fasting, if there is jaundice or if the fluid is infected [4].

Treatment can be conservative and surgical. The goal of conservative management is to decrease lymph production and allow the leak to close. There are two management schemes: fasting and the use of MCT rich formulas to decrease lymph production in the intestine, facilitating closure of the fistula. The success with this treatment has been reported as 63.9% in a series of 103 patients in Japan [5]. Use of octreotide, an analog of somatostatin, has also been reported to decrease lymph production. Very different doses have been used, ranging from 1μg/kg/h to 12μg/kg/h, and there’s no consensus regarding the most effective dose or even how treatment should be started. Karagol et al. reported the management of a patient with feeding based on a formula from 50-80% MCT and octreotide at 4μg/kg/hour without response after 10 days; the patient required fasting and TPN as well as octreotide at 8μg/kg/hour, and the ascites remitted [6]. The time to define whether there is lack of response to medical treatment has not yet been determined. Different articles report a range between 1 and 2 months [5,7,8]. It is important during this period to assure that the patient has good nutrition and rule out hypogammaglobulinemia, two factors associated with infections that could compromise the life of the patient.

Treatment success will depend on etiology, age and nutritional status of the patient, making it difficult to compare management protocols. This case describes the management protocol used at our institution. We initially manage all patients with fasting, parenteral nutrition, and octreotide. Octreotide is begun at 1μg/kg/hour and is increased by 1μg/kg/hr daily up to 10μg/kg/hour, with close surveillance of serum glucose, electrolytes, liver function, thyroid function and the presence of enterocolitis. We usually wait one month with this approach before surgical treatment is considered. If there is a partial response, we can wait another month if the patient does not develop complications. Regarding cholestasis in our patient, it was resolved immediately after drainage of the ascites. It is probable that compression on the bile duct contributed to the cholestasis.

Treatment after discharge should be continued with enteral formula (MCT 85%) (Portagen®). If these formulas are unavailable, Deilem® can be used, externally supplemented with MCT. The latter formula is used at our hospital because in Mexico there are no manufactured formulas high in MCT. This management is continued for 1 or 2 months. A longer period is not advised because central nervous system development requires LCT for adequate development.

## Footnotes

**Source of Support:** Nil

**Conflict of Interest:** Nil
